# Embryonic stem cells and inducible pluripotent stem cells: two faces of the same coin?

**DOI:** 10.18632/aging.100513

**Published:** 2012-12-11

**Authors:** Francesco Romeo, Francesco Costanzo, Massimiliano Agostini

**Affiliations:** ^1^ Department of Experimental and Clinical Medicine, Magna Græcia University of Catanzaro, Salvatore Venuta Campus, 88100 Catanzaro, Italy; ^2^ Medical Research Counsil, Toxicology Unit, University of Leicester, Leicester, LE1 9HN, UK

**Keywords:** embryonic stem cells, inducible pluripotent stem cells, cell-based therapy, self-renewal and differentiation

## Abstract

Embryonic stem cells (ESCs) are derived from the inner cell mass of the blastocysts and are characterized by the ability to renew themselves (self-renewal) and the capability to generate all the cells within the human body. In contrast, inducible pluripotent stem cells (iPSCs) are generated by transfection of four transcription factors in somatic cells. Like embryonic stem cells, they are able to self-renew and differentiate. Because of these features, both ESCs and iPSCs, are under intense clinical investigation for cell-based therapy. In this review, we revisit stem cell biology and add a new layer of complexity. In particular, we will highlight some of the complexities of the system, but also where there may be therapeutic potential for modulation of intrinsic stem cells and where particular caution may be needed in terms of cell transplantation therapies.

## INTRODUCTION

Since the first isolation of human Embryonic Stem Cells (ESCs) [1] huge interest has developed in the scientific and clinical communities and in the public in general because of their therapeutic potential. In particular, attention has focused on their potential use in cell-based therapy for diseases that are refractory to conventional treatments, such as neurodegenerative diseases and immunodeficiency, because of their ability to be programmed into new mature differentiated cells of all lineages [2]. The list of pathologies that in theory can be treated using stem cells includes: Alzheimer's disease [3], Parkinson's disease [4], [5], Huntington's disease [6],stroke [7], diabetes [8], cancer [9], age-related disorders [10-12] haematological disorders [13], cardiovascular disease [14, 15] and bone and muscle regeneration [16]. This interest has further increased in the last 6 years since Takhashi and Yamanaka broke the dogma in developmental biology that mammalian somatic cell differentiation is an irreversible process [17, 18]. By transfection in human somatic cells of four transcription factors (Nanog, Sox2, c-Myc and Klf4) they were able to revert the differentiated cells to an embryonic-like state. Because these newly generated cells show the morphology, pluripotency and capacity to form teratomas like ESC, they named these cells, induced pluripotent stem cells (iPSCs). Thus, this revolutionary step in the field has provided the clinical and scientific communities a second tool for cell-based therapy. Although our knowledge of the molecular mechanisms that control the self-renewal and differentiation of stem cells has grown considerably during the past decade, we still need more basic research in order to understand the molecular mechanisms that regulate proliferation, survival and differentiation of stem cells particularly after transplantation and in the pathological environment.

In this review, we will describe the biology of ESCs and iPSCs, emphasising the common features that they share. We will also review the state of the art of stem cells in clinic.

### ESCs

The following definition of a stem cell is now widely accepted: stem cells are characterized by their ability to renew themselves through mitotic cell division (self-renewal) and the ability to differentiate into diverse range of specialized cell types. In general, stem cells can be classified into two broad types of mammalian cell: 1) embryonic stem cells (ESCs), that are derived from the inner cell mass of the blastocyst, and 2) adult stem cells. Adult stem cells can be found in several tissues and can be further classified into different subtypes. The two groups can be distinguished on the basis of their ability for self-renewal and differentiation. In particular, the self-renewal potential of ESCs is unlimited, and they can generate all the cell types in the body. In contrast, adult stem cells have limited self-renewal and pluripotency, and are important physiologically in tissue repair and homeostasis in the adult.

**Figure 1:**
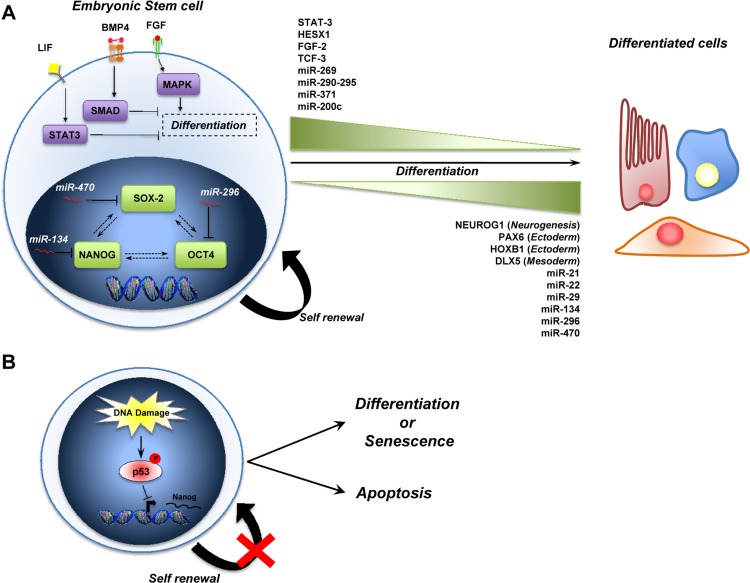
A schematic representation of ESCs biology (**A**) The stemness of ESCs is maintained by intrinsic (i.e. SOX2, NANOG and OCT4) and by extrinsic pathways (i.e. LIF, BMP4 and FGF). MicroRNAs also play a role in the maintenance of stem cells, and some are expressed during self-renewal (*miR-269, miR-290-295 cluster, miR-371, miR-200c*) while others are up-regulated during differentiation (*miR-21, miR-22, miR-29, miR-134, miR-296, miR-470*) (see text for details). **(B)** Role of p53 in the maintenance of genomic stability in ESCs. During DNA damage, p53 is activated (via Ser315 phosphorylation) and binds the Nanog promoter to repress its expression. The outcome of p53 activation is to induce the differentiation of ESCs into other cell types that they can go into a senescent state or induces apoptosis to preserve genome stability.

The genes and the signalling pathways that control the self-renewal and the cell fate decisions is a molecular signature called “stemness” [19]. The cooperation of intrinsic elements (i.e. transcription factors) and extrinsic signals (i.e. leukemia inhibitor factor, bone morphogenetic protein and fibroblast growth factor) from their microenviroment [20-24] regulates the behaviour of stem cells (self-renewal + pluripotency) (Figure 1A). This shows that self-renewal and pluripotency of stem cells is a complex process that requires the coordination of multiple pathways involved in proliferation and the maintenance of an undifferentiated state [21, 22].

**Figure 2:**
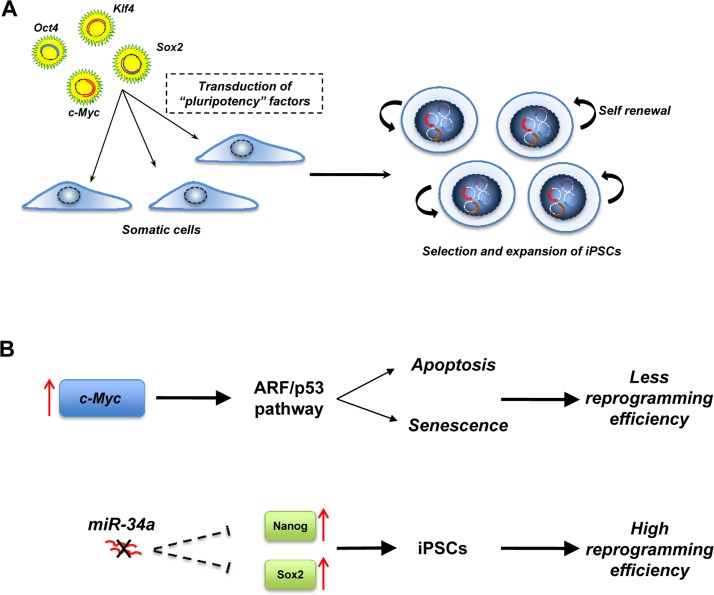
Induced pluripotent stem cells (iPSCs) **(A)** iPSCs are generated by the transduction in somatic cells of “pluripotency” factors. The resulting iPSCs have similar properties to ESCs. **(B)** p53 pathways regulate the efficiency of reprogramming. Overexpression of the oncogenic c-myc during reprogramming induces the ARF/p53 pathway that drives the cells to apoptosis or senescence and the miR-34 family negatively regulates the expression of Nanog and Sox2. In a miR-34 null-context, the expression of Nanog and Sox2 is increased, resulting in a higher reprogramming efficiency.

Three transcription factors, Nanog, Oct4 and Sox2 form the core of a regulatory circuit, which maintains stem cells capacity for self-renewal and pluripotency. Indeed, ESCs deficient in Nanog lose pluripotency and differentiate inappropriately [23, 25]. The POU domain transcription factor, Oct4, is also critical for the pluripotency of ESCs. Oct4 is down regulated during differentiation of ESCs *in vitro* and*in vivo* [26, 27]. Moreover, Oct4 can interact with Sox2 and bind to the Nanog promoter to regulate the expression of Nanog. The SRY-related HMG-box transcription factor Sox2 is also required for the maintenance of pluripotency in ESC *in vivo* and *in vitro* [28-30].

Thus, the Oct4, Sox2 and Nanog transcription factors control the expression of genes, including further transcription factors (such as STAT3, HESX1, FGF-2 and TCF) [31] and other signaling components necessary to maintain the stem cell state. Moreover, they also repress the expression of genes that, if expressed, would promote differentiation (such as NUEROG1, PAX6, HOXB1, DLX5) [32, 33]. This triad also forms an autoregulatory circuit, in which, by binding to their own promoters, as well as to the promoters of Oct4, Sox2 and Nanog they collaborate to sustain their expression.

Among the extrinsic factors, LIF (Leukemia Inhibitor factor) blocks the differentiation of mouse ESCs through the binding to its receptor and subsequent activation of Jak/STAT3 signaling [34, 35]. Activation of this pathway is essential for self-renewal of ESC and is necessary to maintain the undifferentiated state of ESCs[36]. Another extrinsic factor that is critical for the maintenance of the pluripotency of ESCs is bone morphogenetic protein 4 (BMP4) [37, 38]. BMP4 binds the BMP receptor and activates SMAD proteins, which in turn promote the expression of inhibitor of differentiation (Id) proteins. The Id proteins block lineage commitment and permit self-renewal of ESCs [39, 40], for example by blocking the ESC differentiation induced by Fibroblast Growth Factor (FGF) via MAPK signaling [41].

Recently, several observations indicate that the p53 family is involved in the regulation of stem cell biology [42]. The first indication of a direct p53 involvement in this process comes from the observation that p53 regulates Nanog expression [43, 44]. In particular, it has been shown that after induction of DNA damage in mouse embryonic stem cells (mESCs), p53 is phosphorylated at Ser315 and binds to the promoter of *Nanog*, suppressing its expression. Consequently, the result of p53 activation in this system is to bring mESCs into a more differentiated state where the cells can efficiently undergo p53-dependent cell cycle arrest or apoptosis, promoting the preservation of genomic stability. This role of p53 is supported by the fact that the loss of p53 in human ESCs leads to an increase of genomic instability [45]. Moreover, p53 participates in the control of neural stem cells (NSC). Loss of p53 leads to an elevated proliferation rate as well as an increase in the self-renewal of *in vitro* propagated p53−/− neural stem cells [46], although the precise contribution of p53 to NSC differentiation is somewhat controversial [47].

**Figure 3:**
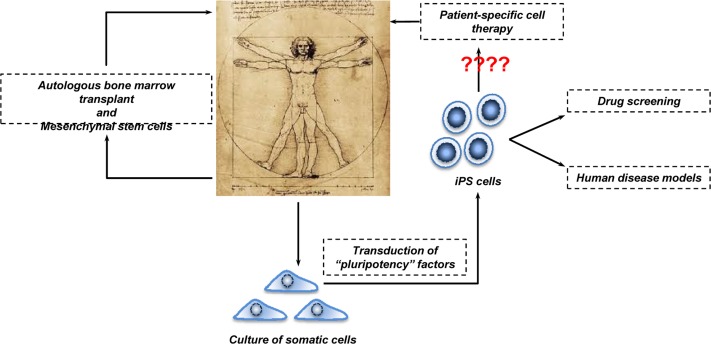
Human cell-based therapy Bone marrow transplantation is widely employed in the clinic for several diseases including cancer and haematological disorders. More recently, MSCs are stepping in. They could be used to treat inflammatory conditions such as Multiple Sclerosis and Pulmonary Fibrosis. The employment of iPSCs in cell-based therapy is a relative new tool. However, because their potential tumorigenicity in the clinical setting remains to be clarified, perhaps iPSCs should stay on the bench meanwhile and be used in drug screening and human disease models.

Another p53 family member, p73 [48-52] is also required for the maintenance of NSCs. Indeed, several experimental findings demonstrate that p73, in particular the TAp73 isoform, is a positive regulator of embryonic and adult NSCs. p73 null mice show a reduction in neurogenesis in the subgranular zone of the dentate gyrus and in the subventricular zone, and neurospheres derived from p73 null mice grow more slowly and are smaller. The potential downstream candidates responsible for this phenotype are genes involved in the regulation of proliferation and/or self-renewal pathways [24, 53], and loss of p73 leads to a transcriptional deregulation of *Sox-2, Sox-3, Nanog, Notch-I, Notch-2, Hes-5, Jag2, Hey-2* and *Deltex*.

With the discoveries of microRNAs (miRs), a new layer of complexity has been added to stem cell biology [54, 55]. miRs (for instance miR-269, the miR-290-295 cluster, miR-371 and miR-200c), have been found to be preferentially expressed in undifferentiated stem cells and their expression levels decrease as the stem cells differentiate [56]. In contrast, the expression of other miRs (miR-21, miR-22 and miR-29) increases during the differentiation of ESCs indicating a possible role for them in stem cell differentiation [57, 58]. Moreover, loss of the components of the miR processing machinery, such as Dicer and Drosha-DGCR8-Ddx5 affect both self-renewal and differentiation of ESCs [59, 60]. The Nanog-Oct4-Sox2 triad is also a target of miRs during the differentiation of ESCs. In particular, miR-134, miR-296 and miR-470 are up regulated during the differentiation of mouse embryonic stem cells induced by retinoic acid, and target these three transcription factors. This leads to transcriptional and morphological changes characteristic of differentiating mouse ESCs. More recently, miR-124 and miR-34a have been shown to contribute to neuronal differentiation of ESCs [61-63].

### iPSCs

Nuclear transplantation, cellular fusion and cell explantation are the strategies that scientists have employed in order to induce the conversion of differentiated cells into an embryonic state [17]. However, starting from the “simple” assumption that the factors that play a key role in the maintenance of pluripotency of ESC could work as reprograming factors, Yamanaka and colleagues were able to reprogram somatic cells into pluripotent ESCs [17]. They first identified genes that were highly and specifically expressed in the ESC, but not in somatic cells, and divided these into three groups. The first group comprised genes responsible for the maintenance of a pluripotent state (Nanog, Sox2 and Oct3/4). The second group contained c-Myc, Stat-3 and TCl1, tumor-associated genes. Finally, the third group contained genes that have a specific role in ESCs such as Kfl-4, ECAT1 and Esg1. Different combinations of genes in the three groups were transfected into mouse embryonic fibroblasts with the result that a combination of only four factors (Oct4, Sox2, Klf4 and c-Myc), were sufficient to reprogram somatic cells into iPSCs. While Oct-4 and Sox-2 are required for reprogramming, a combination of other factors, such as Oct4, Sox2, Nanog and Lin28, is also able to reprogram somatic cells into iPSCs [64]. However, Nanog is dispensable, although two oncogenic factors (c-Myc and Klf4) are essential for reprogramming. Phenotypically, the iPSC are similar to ESC in many aspects, including morphology, surface markers, gene expression, *in-vitro* differentiation and teratoma formation when they are injected in immunocompromised mice. However, the precise molecular mechanism of reprogramming remains unclear. While the role of Oct3/4 and Sox2 could be predicted by the fact that both have a role in the control of pluripotency in ESCs, the exact role of c-Myc and Klf4 remains to be clarified. We can speculate that c-Myc and Klf4 act as modifiers of chromatin structure allowing Oct3/4 and Sox2 to bind their target genes that are normally silenced by epigenetic mechanisms in differentiated cells.

While the ability to develop iPSCs from differentiated somatic cells is exciting, the system has two major drawbacks. Firstly, the reprogramming efficiency is very low, suggesting that inside the cell there may be mechanisms that prevent the reprogramming process: secondly, there is the oncogenic potential of iPSCs, as reflected in their ability to form teratomas in mice. Several findings suggest that p53 is responsible for the low efficiency in the reprogramming of somatic cells [65]. Indeed, overexpression of the oncogene c-Myc induces the ARF/p53 pathway driving the cells towards apoptosis or senescence [66]. This is also supported by the fact that the efficiency of reprogramming is higher in a p53 null context [67-71]. Recently, some observations indicate that the miR-34 family [72] may also regulate reprogramming of somatic cells, and deficiency of miR-34 significantly increases reprogramming efficiency and kinetics [73]. In contrast to p53 deficiency, which enhances reprogramming at the expense of iPSC pluripotency, genetic ablation of miR-34 promoted iPSC generation without compromising self-renewal or pluripotency. Suppression of reprogramming by miR-34a was due, at least in part, to repression of pluripotency genes, including Nanog, Sox2 and N-Myc. However, miR-34a ablation only partially phenocopies that of p53 [68] (and combined deletion of miR-34a and p21 similarly fails to reproduce the p53−/− phenotype), suggesting that p53 provides a barrier to iPSC generation through mechanisms and targets not fully characterized. Because inactivation of p53 and/or downstream components of its pathway seem to be critical for reprogramming efficiency, this raises concern about the tumorigenicity of iPSCs, since chromosomal aberrations have been found in iPSCs[74]. Moreover, injection of iPSCs into blastocysts led to an incidence (about 20%) of tumors in the resultant chimeric mice, attributable to reactivation of the c-Myc transgene [77].

### Stem Cells in Therapy: myth or reality

As mentioned above, cell-based therapy has generated a lot of excitement in the scientific and clinical communities. In order to understand how far or close we are from the reality, we scanned the public clinical trials database of the National Institute of Health. Typing “stem cells” into http://clinicaltrials.gov database revealed that there are more than 4000 clinical trials in progress at different stages (Recruiting, Phase I/II and Terminated) for a wide range of diseases (reviewed in [76]). Overall, the following picture emerges. First, extensive experience, extending over more than 30 years, on the transplantation of bone marrow hematopoietic stem cells into patients affected by blood pathologies and cancer provides reasonable confidence on the safety and efficacy of cell-based therapy.

A second message is the use of mesenchymal stem cells (MSCs) as alternatives to the stem cell sources described above. MSCs are adult stem cells that are traditionally found in the bone marrow. However, they can be also isolated from other tissues such as cord and peripheral blood. MSCs can be differentiated to form adipocytes, cartilage, bone, muscle and skin, and therefore are under consideration for bone and cartilage repair and autoimmune disease [77]. One advantage of using MSCs in cell-based therapy is the fact that MSCs are of low immunogenicity (due to lack of surface MHC class II expression) and thus well tolerated during transplantation. However, since it has been shown that MSCs support tumor growth in allogenic animals, more basic research is essential in order to better understand the potential and especially the limits of MSCs [78-82].

The alternative to the use of MSCs, and one probably of broader clinical applicability, is to use iPSCs [83]. Like MSCs, these are autologous, and the problem of immunological rejection and use of potent immunosuppressants implicit in ESC therapy does not arise. However, although iPSCs largely resemble ESCs with respect to gene expression profile, epigenetic signature and differentiation capability, their use as therapy is clouded by their potential tumorigenicity. In particular, there is recent evidence reprogramming into iPSCs induces genetic and epigenetic abnormalities [84-89], including copy number variation, point mutations, DNA methylation and alteration of chromosome numbers. The clinical use of iPSCs must therefore proceed with caution. Nevertheless, several studies have already demonstrated that it is possible to generate iPSCs from somatic cells of patients affected by diabetes[90], neurodegenerative diseases [91-93] and psychiatric disorders such as schizophrenia [94]. The generated iPSCs recapitulate *in vitro* much of the cellular pathology of the disease, and, quite apart from any therapeutic potential, could be useful for more detailed analysis of disease pathology [95, 96], drug screening and associated toxicology.

## Abbreviations

ESCs: Embryonic stem cells
iPSCs: inducible pluripotent stem cells
MSC: Mesenchymal stem cells
Nanog: Homeobox transcription factor
Oct4: Octamer3/4
Sox2: SRY box-containing gene 2
Klf4: Kruppel-like factor 4
LIF: Leukemia Inhibitor Factor
BMP: Bone Morphogenetic Protein
FGF: Fibroblast Growth Factor
miR: microRNA
